# SAMHD1 enhances nucleoside-analogue efficacy against HIV-1 in myeloid cells

**DOI:** 10.1038/srep42824

**Published:** 2017-02-21

**Authors:** Paula Ordonez, Simone Kunzelmann, Harriet C. T. Groom, Melvyn W. Yap, Simon Weising, Chris Meier, Kate N. Bishop, Ian A. Taylor, Jonathan P. Stoye

**Affiliations:** 1 Retrovirus-Host Interactions Laboratory, The Francis Crick Institute, London, UK; 2 Structural Biology Science Technology Platform, The Francis Crick Institute, London, UK; 3 Infection and Replication of Retroviruses Laboratory, The Francis Crick Institute, London, UK; 4 Organic Chemistry, Department of Chemistry, Faculty of Sciences, University of Hamburg, Germany; 5 Macromolecular Structure Laboratory, The Francis Crick Institute, London, UK; 6 Faculty of Medicine, Imperial College London, London, UK

## Abstract

SAMHD1 is an intracellular enzyme that specifically degrades deoxynucleoside triphosphates into component nucleoside and inorganic triphosphate. In myeloid-derived dendritic cells and macrophages as well as resting T-cells, SAMHD1 blocks HIV-1 infection through this dNTP triphosphohydrolase activity by reducing the cellular dNTP pool to a level that cannot support productive reverse transcription. We now show that, in addition to this direct effect on virus replication, manipulating cellular SAMHD1 activity can significantly enhance or decrease the anti-HIV-1 efficacy of nucleotide analogue reverse transcription inhibitors presumably as a result of modulating dNTP pools that compete for recruitment by viral polymerases. Further, a variety of other nucleotide-based analogues, not normally considered antiretrovirals, such as the anti-herpes drugs Aciclovir and Ganciclovir and the anti-cancer drug Clofarabine are now revealed as potent anti-HIV-1 agents, under conditions of low dNTPs. This in turn suggests novel uses for nucleotide analogues to inhibit HIV-1 in differentiated cells low in dNTPs.

Sterile α-motif/histidine-aspartate domain-containing protein 1 (SAMHD1) is an antiretroviral protein that restricts HIV-1 infection in non-cycling cells such as macrophages^1^, dendritic cells (DCs)^2^ and resting CD4^+^ T-cells^3^,^4^. Viruses from the HIV-2/SIV_smm_ and SIV_rcm_/SIV_mnd-2_ lineages encode the accessory protein Vpx that overcomes this restriction by directing SAMHD1 for proteasomal degradation^1^,^2^,^5^,^6^.

The prevailing hypothesis is that SAMHD1 restricts HIV-1 replication through its dNTP triphosphohydrolase activity by depleting the intracellular dNTP pool to levels that do not support viral reverse transcription^7^,^8^,^9^,^10^. More recently, it has been proposed that SAMHD1 nucleic acid binding and a nuclease activity might contribute to alternative mechanisms of restriction^11^,^12^,^13^,^14^. However, although measurements of nucleic acid binding support this notion^14^,^15^, variability in the *in vitro* measurements of nuclease activity appear inconsistent with this idea^11^,^14^. In contrast, the nature of the allosteric regulation of SAMHD1 triphosphohydrolase activity through nucleotide binding and tetramerisation has been extensively characterised both structurally^10^,^16^,^17^,^18^,^19^ and biochemically^19^,^20^,^21^,^22^.

SAMHD1 restriction activity is also regulated by phosphorylation. In cycling THP_1 cells that are relatively permissive to HIV-1 infection, SAMHD1 is largely phosphorylated by cyclin A2/CDK1 at Threonine 592. By contrast, T592 phosphorylation is reduced in differentiated THP-1 cells that are restrictive to HIV-1 infection^23^,^24^,^25^. In other cell types and primary macrophages, CDK2 has been proposed to be the kinase that phosphorylates SAMHD1^26^,^27^ controlled by upstream regulation through the cyclin D3/CDK6 complex^28^,^29^. Moreover, CyclinL2 has been proposed to be a negative regulator of SAMHD1 in macrophages^30^, whereas a cyclin D2/CDK4/p21 complex has been proposed to be responsible for maintaining the non-phosphorylated form of SAMHD1 in GM-CSF derived macrophages^31^.

The rate of HIV-1 proviral synthesis is limited by the intracellular dNTP concentration^32^ and it can be accelerated in non-dividing cells by elevating intracellular dNTP levels^33^. Although SAMHD1 reduces the dNTP pool in non-cycling cells thereby decreasing HIV-1 infection^7^,^8^,^9^, other reports showed that SAMHD1 depletion of dNTP levels in cells could also increase the susceptibility of HIV-1 to nucleoside reverse transcriptase inhibitors (NRTIs) used in antiretroviral therapy, likely by decreasing the levels of dNTPs that can compete with chain terminators during proviral synthesis^34^,^35^,^36^.

Agents that modulate SAMHD1 function would have great value for studies of its anti-HIV effects. Since the triphosphohydrolase activity of SAMHD1 is regulated allosterically by nucleotide analogues whilst the efficacy of nucleotide analogues can be affected simultaneously by SAMHD1 activity, we used a combination of *in vitro* and cell-based assays to study the mutual effects of nucleotide analogues and SAMHD1 activity on HIV-1 replication.

We first used an *in vitro* enzyme-coupled assay to test the effect on SAMHD1 activity of the triphosphate derivatives of a panel of FDA-approved nucleoside analogues widely used in antiviral and anticancer therapy, detailed in Table 1. Aciclovir (ACV) and Ganciclovir (GCV) are acyclic guanosine analogues used as anti-herpesvirus agents^37^,^38^,^39^. The halogenated adenosine analogue Clofarabine (CFB) is employed in anticancer therapy^40^,^41^. The NRTIs Stavudine (d4T)^42^,^43^, Didanosine (ddI)^44^ and Abacavir (ABC)^45^ are selective inhibitors of HIV-1 and HIV-2 replication used in HIV/AIDS therapy^46^,^47^. We next tested whether the presence of SAMHD1 caused changes in the anti-HIV-1 efficacy of these nucleoside analogues in phorbol myristate acetate (PMA)-treated and untreated human monocytoid cell lines. We also compared the efficacy of nucleoside analogues in U937 cells expressing SAMHD1 or the catalytically inactive mutant HD206–7AA, and in THP-1 cells expressing endogenous SAMHD1 or transduced with Vpx. Surprisingly, this analysis revealed anti-HIV-1 activities for ACV, GCV and CFB in addition to the NRTIs in PMA-treated cells; these were further enhanced in the presence of added SAMHD1.

## Results

### *In vitro* activity of nucleotide analogues

**Figure 1:**
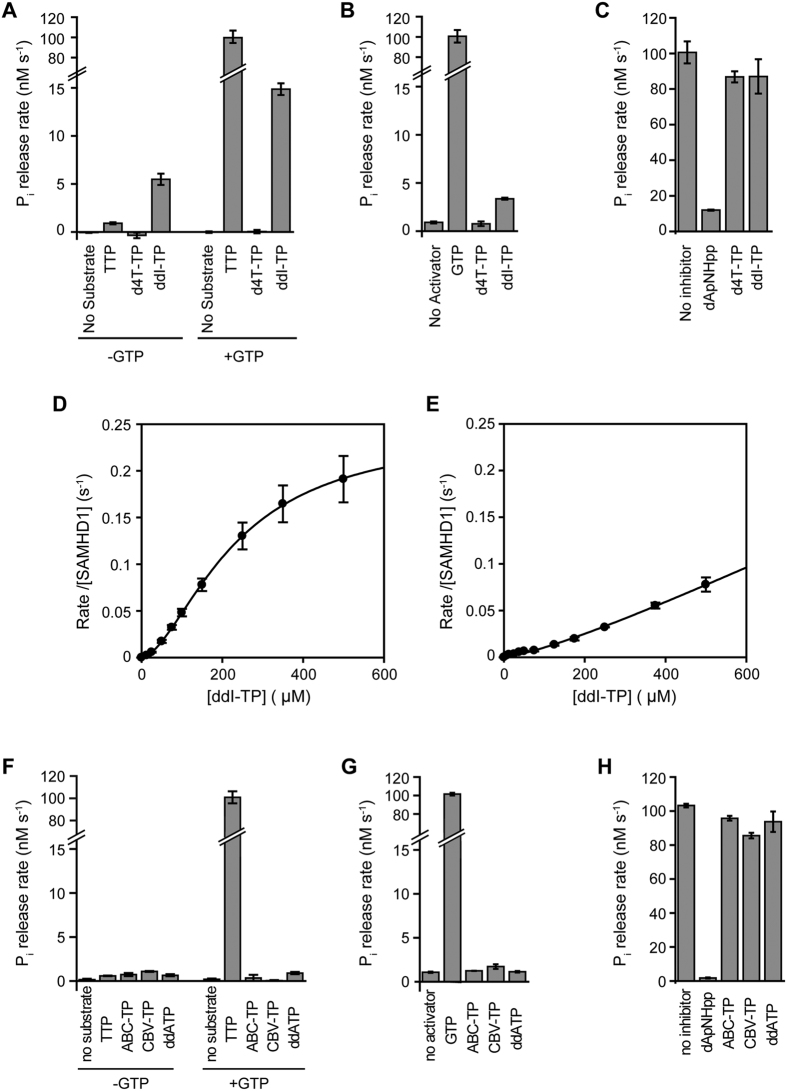
The effects of d4T-TP,ddI-TP,ddA-TP, ABC-TP and CBV-TP on SAMHD1 triphosphohydrolase activity. (**A**) Hydrolysis of 0.3 mM TTP, d4T-TP or ddI-TP in the absence (left) and presence (right) of 0.1 mM activator GTP. (**B**) Activation of SAMHD1 TTP hydrolysis. Triphosphohydrolase activity was measured with 0.3 mM TTP substrate upon addition of 0.1 mM GTP, d4T-TP or ddI-TP as activators. (**C**) Inhibition of SAMHD1 GTP-activated TTP hydrolysis. Triphosphohydrolase activity was measured with 0.3 mM TTP and 0.1 mM GTP activator alone and with addition of 0.3 mM dApNHpp positive control, d4T-TP or ddI-TP. Error bars are the standard error of the mean (SEM) of three independent measurements. (**D**) Concentration dependence of SAMHD1 ddI-TP hydrolysis in the presence of 0.2 mM GTP (saturating activator concentration). Nonlinear least squares fitting using a Hill equation gives the apparent binding constant *K*_*S*_ = 226 ± 12 μM, catalytic constant *k*_*cat*_ = 0.24 ± 0.04 s^−1^ and the Hill coefficient n = 1.7 ± 0.1 (Mean ± SEM). (**E**) ddI-TP allosteric activation of TTP hydrolysis. Rates were determined for 1 mM TTP at varying dd-ITP concentration. Nonlinear least squares fitting gives only lower estimates for the maximal rate and the ddI-TP concentration at half maximal activation of *k*_*max*_ > 0.1 s^−1^ and *K*_*a*_ > 300 μM. (**F**) Hydrolysis of 0.3 mM TTP, ABC-TP, CBV-TP and ddATP in the absence (left) and presence (right) of 0.1 mM activator GTP. (**G**) Activation of SAMHD1 TTP hydrolysis. Triphosphohydrolase activity was measured with 0.3 mM TTP substrate upon addition of 0.1 mM GTP, ABC-TP, CBV-TP or ddATP as activators. (**H**) Inhibition of SAMHD1 GTP-activated TTP hydrolysis. Triphosphohydrolase activity was measured with 0.3 mM TTP and 0.1 mM GTP activator alone and with addition of 0.3 mM dApNHpp positive control, ABC-TP, CBV-TP or ddATP. Error bars are the range of data from two independent measurements.

Since the triphosphohydrolase activity of SAMHD1 is allosterically regulated by nucleotide analogues, and nucleotide analogues can also be hydrolysed by SAMHD1, we used a coupled-enzyme *in vitro* assay to assess their role as activators, substrates or inhibitors of SAMHD1. We evaluated the triphosphate forms of the panel of nucleoside analogues detailed in Table 1 as well as dideoxyguanosine triphosphate (ddATP) and Carbovir triphosphate (CBV-TP) that are active antiretroviral agents produced after cellular conversion of ddI to ddATP^48^,^49^ and ABC to CBV-TP^50^,^51^. Our previous *in vitro* assays, demonstrated that Aciclovir triphosphate (ACV-TP) was a non-hydrolysable allosteric activator of SAMHD1 with comparable efficiency to GTP, Ganciclovir triphosphate (GCV-TP) was also non-hydrolysable but displayed no activation of SAMHD1 and Clofarabine triphosphate (CFB-TP) was a substrate hydrolysed by SAMHD1 at a rate comparable to natural dNTP substrates in the presence of the activator GTP^21^. Other studies have demonstrated SAMHD1 has little or no hydrolytic activity against stavudine triphosphate (d4T-TP)^35^,^36^. Our data confirm this observation (Fig. 1A) and in addition demonstrate that d4T-TP is not a SAMHD1 inhibitor or activator (Fig. 1B,C). By contrast, didanosine triphosphate (ddI-TP) is hydrolysed by SAMHD1 even in the absence of GTP. In the presence of activating GTP, ddI-TP hydrolysis increases three-fold, to around six-fold slower than the TTP hydrolysis rate (Fig. 1A). In addition, ddI-TP activates SAMHD1 triphosphohydrolase activity, but to a much lesser degree than the natural activator, GTP (Fig. 1B) and displays no significant inhibitory effects (Fig. 1C). Quantification of ddI-TP hydrolysis data, demonstrates cooperativity with respect to ddI-TP concentration (Fig. 1D). Data are best fit with a Hill equation with parameters *K*_*S*_ = 226 ± 12 μM, n = 1.7 ± 0.1 and *k*_*cat*_ = 0.24 ± 0.04 s^−1^. ddI-TP only weakly activates SAMHD1 and analysis of the ddI-TP concentration dependence of TTP hydrolysis (Fig. 1E) yields only lower estimates for the maximal rate and the ddI-TP concentration at half maximal activation, *k*_*max*_ > 0.1 s^−1^ and *K*_*a*_ > 300 μM. By contrast, analysis of ddATP, the cellular active form of ddI, revealed no hydrolysis by SAMHD1, as has been shown previously^34^,^35^ and additionally there was no measurable activation or inhibition of SAMHD1 by ddATP (Fig. 1F–H). Examination of Abacavir triphosphate (ABC-TP) and Carbovir triphosphate (CBV-TP) also showed they were not hydrolysed by SAMHD1 and neither ABC-TP nor CBV-TP displayed any activation or inhibition of SAMHD1 (Fig. 1F–H).

### Anti-HIV-1 activities of nucleoside analogues

The anti-HIV-1 activities of the six selected nucleoside-analogues and non-nucleoside RT inhibitor (NVP) and integrase inhibitor (RAL) controls were evaluated in proliferating H9 and Jurkat T cell lines (Supplementary Table S1). As would be expected for T cell lines, the anti-herpesvirus agents ACV and GCV showed no anti-HIV-1 activity when tested at concentrations up to 200 μM. The anticancer drug CFB did have anti-HIV-1 activity with EC_50_ values of 0.094 and 0.663 μM in H9 and Jurkat cells, respectively. The three NRTIs (d4T, ddI and ABC) showed anti-HIV-1 activities with EC_50_ values below 10 μM; NVP and RAL inhibited HIV-1 at nanomolar concentrations (Supplementary Table S1).

**Figure 2:**
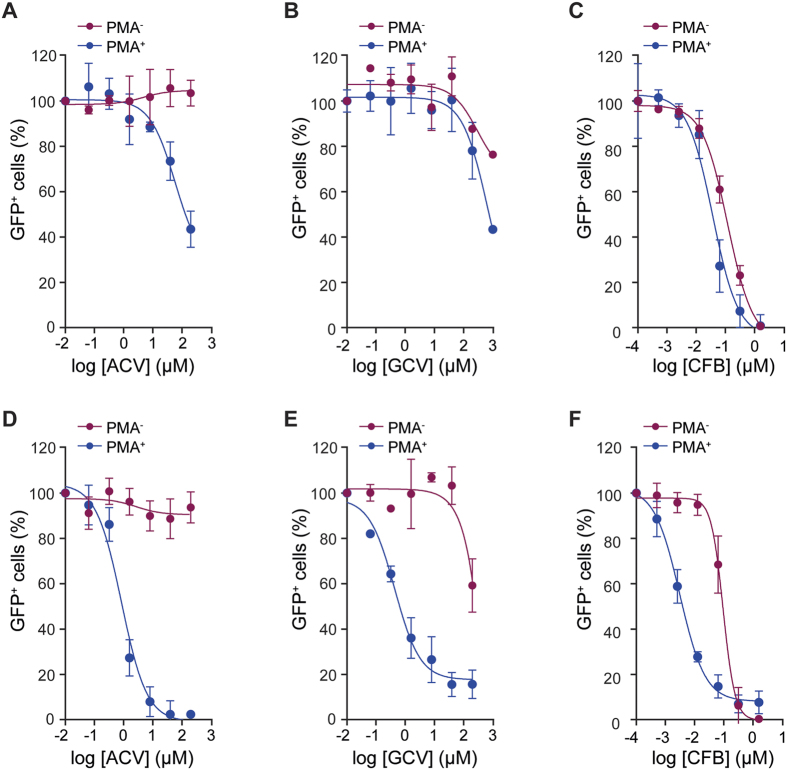
Anti-HIV-1 activities of ACV, GCV and CFB in monocytoid cell lines. PMA^−^ (red) and PMA^+^ (blue) U937 (**A**–**C**) and THP-1 (**D**–**F**) cells were cultured in the presence of increasing concentration of Aciclovir (ACV) (**A**,**D)**, Ganciclovir (GCV) (**B**,**E**) and Clofarabine (CFB) (**C**,**F**). Cells were incubated with HIV-1-GFP and the percentage of infected GFP^+^ cells, measured by flow cytometry. To account for differences in overall infectivity of PMA^−^ and PMA^+^ cells, in each panel the data is plotted as the normalised percentage of GFP^+^ cells against drug concentration. Error bars represent the standard deviation from at least two independent experiments.

By contrast, when the anti-HIV-1 effects of these compounds were assessed in undifferentiated (PMA^−^) and PMA-differentiated (PMA^+^) U937 and THP-1 lines a different pattern emerged (Table 2). Here, although the anti-herpesvirus agents ACV and GCV did not show anti-HIV-1 activity in PMA^−^ cells (EC_50_ > 200 μM), ACV showed an increased activity in PMA^+^ U937 cells (EC_50_ = 52.2 μM), and both ACV and GCV had extremely significant activities in PMA^+^ THP-1 cells (EC_50_ 0.657 and 0.367 μM; *p* values < 0.0001) (Fig. 2A,B,D,E). The anti-HIV-1 activity of CFB also increased in PMA^+^ cells. In U937, the increase was 3-fold and in THP-1 cells the increase was highly significant at 44-fold (EC_50_ = 0.003 μM) (Fig. 2C,F). Measurements of the established NRTIs also revealed increases of anti-HIV-1 activities in PMA^+^ cells, with the greatest effects observed in THP-1 cells. Thus, ddI and ABC had greatly increased anti-HIV-1 activities in PMA^+^ cells, down to nanomolar EC_50_ in THP-1 cells (22- and 16-fold, respectively) (Table 2). By contrast, d4T had similar anti-HIV-1 activity in PMA^+^ and PMA^−^ U937 cells, with only a small increased activity (2-fold) when comparing PMA^+^ to PMA^−^ THP-1 cells (Table 2). As might be expected, the NVP and RAL controls showed only minor, non-significant differences (*p* values > 0.005) in their activities in PMA^−^ and PMA^+^ cells (Table 2).

Increased anti-HIV-1 activities of NRTIs have been reported previously in PMA^+^ treated THP-1 cells and monocyte-derived macrophages (MDM), where dNTP levels are reduced^34^,^35^,^36^. However, our observations reveal that compounds with no apparent anti-HIV-1 activity in T-cells or undifferentiated monocytoid cell lines (ACV and GCV) can display marked anti-HIV-1 effects in differentiated monocytoid cells. Moreover, this observation is particularly marked in the differentiated THP-1 cells where SAMHD1 is expressed in its highly active non-phosphorylated form^24^.

### Cytotoxicity of nucleotide analogues

To compare the anti-HIV-1 activities and cytotoxic effects of the nucleoside analogues in T- and monocytoid cell lines, we determined the cytotoxic concentration (CC_50_) by flow cytometry and calculated the selectivity index (SI) for each compound (Supplementary Tables S1 and S2). With the exception of CFB, all nucleoside analogues displayed no toxicity in any of the cell lines when evaluated at concentrations up to 200 μM (CC_50_ > 200 μM). In T cells, where ACV and GCV showed no anti-HIV-1 activity they also did not exhibit any cytotoxic effect (CC_50_ > 200 μM). The anti-HIV-1 activity of CFB in T cells was only observed at greater than cytotoxic concentrations (SI < 1). In contrast, NRTIs (d4T, ddI, ABC) showed SIs > 30 (Supplementary Table S1).

In monocytoid U937 and THP-1 cells, SI values increased significantly upon differentiation (Supplementary Table S2). ACV showed SI values of >4 and >304 in PMA^+^ U937 and THP-1 cells, respectively. Similarly, the SI of GCV increased to >545 in PMA^+^ THP-1 cells. As observed in T cells, the anti-HIV-1 activity of CFB in PMA^−^ cells was only observed at greater than cytotoxic concentrations (SI < 1). However, upon differentiation cytotoxicity was greatly decreased (CC_50_ > 1.6 μM) resulting in SI values of >43 and >533 in PMA^+^ U937 and THP-1 cells, respectively. The NRTIs (d4T, ddI, ABC) did not show cytotoxicity (CC_50_ > 200 μM) thus increasing the SIs, particularly in PMA^+^ THP-1 cells (Supplementary Table S2).

### Effect of SAMHD1 on the anti-HIV-1 activity of nucleoside analogues

To test whether the anti-HIV-1 activities of the selected nucleoside analogues could be further increased through dNTP depletion by SAMHD1, we evaluated antiviral activities in undifferentiated (PMA^−^) and differentiated (PMA^+^) U937 cells transduced with catalytically active SAMHD1 (*wt*) or an inactive mutant (HD206-7AA). Comparative EC_50_ data and statistical significance test results from these assays are summarised in Table 3.

**Figure 3:**
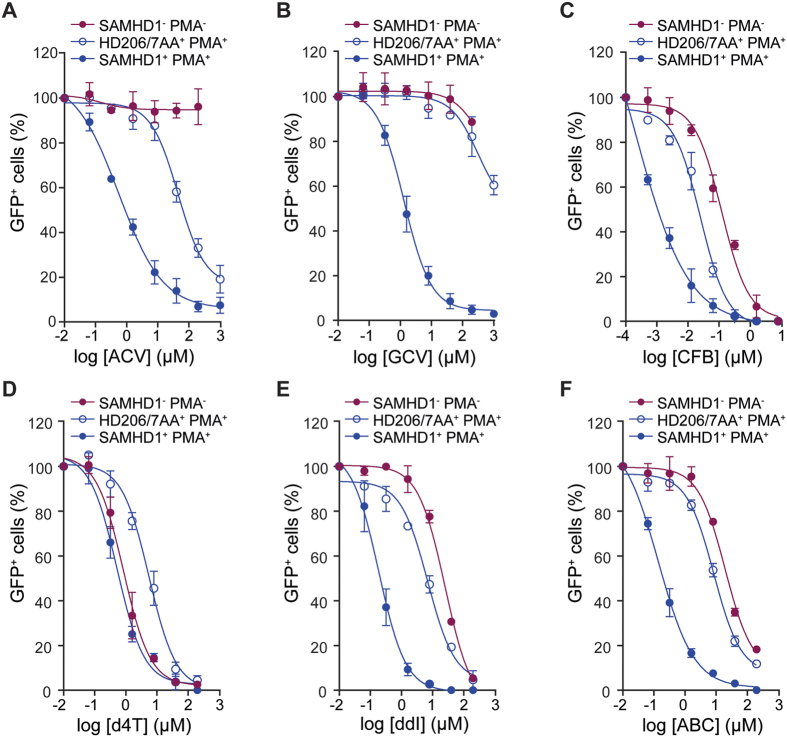
Anti-HIV-1 activities of nucleoside analogues in U937 cells expressing SAMHD1. Antiviral activities were determined in undifferentiated (PMA^−^) and differentiated (PMA^+^) U937 cells expressing SAMHD1 or the mutant HD206-7AA. Cells were cultured in the presence of increasing concentration of Aciclovir (ACV) (**A)**, Ganciclovir (GCV) (**B**), Clofarabine (CFB) (**C**), Stavudine (d4T) **(D)**, Didanosine (ddI) **(E)** and Abacavir (ABC) **(F)**. Cells were incubated with HIV-1-GFP and the percentage of infected GFP^+^ cells was measured by flow cytometry. PMA^−^ cells expressing SAMHD1 (red filled circles), PMA^+^ cells expressing the mutant HD206-7AA (blue open circles) and PMA^+^ cells expressing SAMHD1 (blue filled circles) are shown. Infectivity data is plotted as the normalised percentage of GFP^+^ cells against drug concentrations. Error bars represent the standard deviation from at least 2 independent experiments.

Inspection of these data showed that ACV and GCV had no anti-HIV-1 activity in PMA^−^ cells transduced with either *wt* SAMHD1 or HD206-7AA. In PMA^+^ cells expressing HD206-7AA, ACV had anti-HIV-1 activity with EC_50_ = 50.9 μM (Fig. 3A) comparable to that observed in untransduced U937 PMA^+^ cells (Fig. 2A). However, in PMA^+^ cells expressing *wt* SAMHD1, ACV anti-HIV-1 activity was enhanced a further 78 fold (EC_50_ = 0.652 μM; *p* < 0.0001) (Fig. 3A). Similarly, GCV had no significant anti-HIV-1 activity in PMA^+^ cells expressing HD206-7AA (EC_50_ > 200 μM) but in cells expressing *wt* SAMHD1 anti-HIV-1 activity of GCV was also greatly enhanced, >200 fold (EC_50_ = 0.876 μM; *p* < 0.0001) (Fig. 3B). As was observed for endogenous SAMHD1 expression in THP-1 cells, these data demonstrate that exogenous expression of SAMHD1 in other differentiated monocytoid cells also greatly enhances the anti-HIV activities of ACV and GCV anti-herpesvirus agents.

Analysis of CFB data showed transduction of *wt* SAMHD1 or HD206-7AA did not affect the EC_50_ values of CFB in PMA^−^ cells, which remained higher than the cytotoxic concentrations (Supplementary Table S2, SI < 1). Upon introduction of HD206-7AA into PMA^+^ U937 cells a small reduction in the CFB EC_50_ was observed (Fig. 3C) but only to a level comparable with untransduced PMA^+^ U937 cells (Table 2). In contrast, there was a 22-fold increase in the anti-HIV-1 activity of CFB in PMA^+^ cells expressing *wt* SAMHD1 over those expressing HD206-7AA (Fig. 3C). Although, not as large as the effects observed with ACV and GCV these data demonstrate that whilst differentiation of U937 cells alone reduces the EC_50_ of CFB to below cytotoxic levels, additional expression of SAMHD1 in differentiated monocytoid cells enhances the anti-HIV activity of CFB even further.

The NRTIs d4T and ddI showed only slightly greater anti-HIV-1 activities in PMA^−^ cells expressing *wt* SAMHD1 over those transduced with HD206/7AA (3- and 2-fold, respectively) and ABC showed no significant difference (Table 3). In PMA^+^ HD206-7AA expressing cells the antiviral activities of all NRTIs (d4T, ddI and ABC) were increased but only to EC_50_ values comparable with those observed in untransduced cells (Table 2). However, in PMA^+^ cells expressing *wt* SAMHD1 the antiviral activities of all NRTIs (d4T, ddI and ABC) were increased significantly (11-, 81- and 41-fold, respectively) compared to cells expressing HD206-7AA (Fig. 3D–F). These data support the notion that SAMHD1 specifically enhances the efficacy of NRTIs through triphosphohydrolase activity acting on the cellular dNTP pool. This argument is strengthened further by the observations that the non-nucleoside RT inhibitor NVP and integrase inhibitor RAL did not show any significant difference in anti-HIV-1 activities upon *wt* SAMHD1 expression in PMA^−^ or PMA^+^ cells (Table 3).

### Vpx knockdown of SAMHD1 activity

Since we found significant differences in the anti-HIV-1 activities of nucleoside analogues in U937 cells expressing exogenous *wt* SAMHD1 or HD206-7AA, we further evaluated their anti-HIV-1 activities in undifferentiated (PMA^−^) and differentiated (PMA^+^) THP-1 cells expressing endogenous SAMHD1 after transduction with the HIV-2 accessory viral protein Vpx, which reduces SAMHD1 levels by targeting it for proteasomal degradation^1^,^2^,^5^,^6^. These data and statistical significance test results are summarised in Table 4.

**Figure 4:**
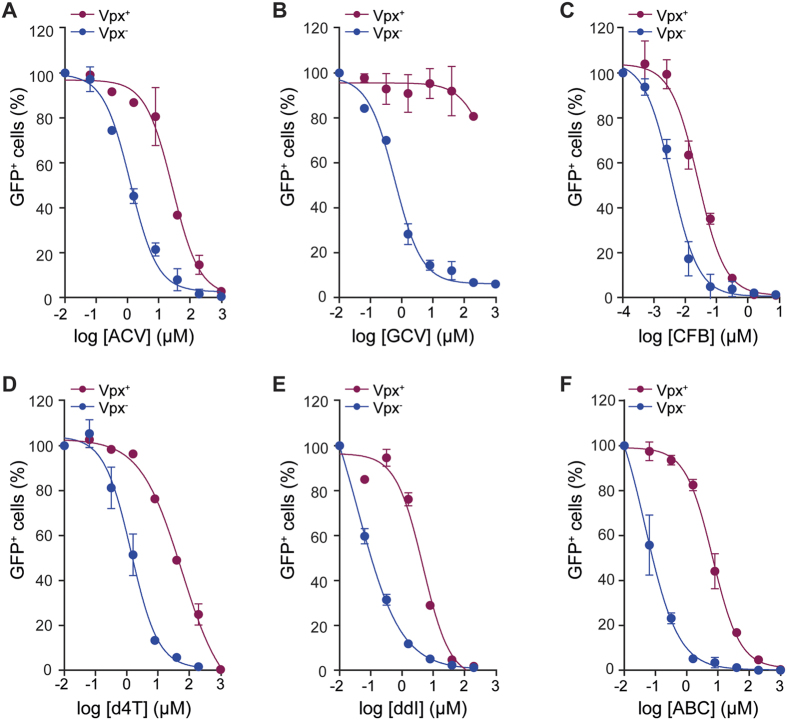
Anti-HIV-1 activities of nucleoside analogues in differentiated THP-1 cells after Vpx knockdown of endogenous SAMHD1 expression. Antiviral activities were determined in differentiated (PMA^+^) THP-1 cells expressing endogenous SAMHD1 and after transduction with Vpx. Cells were cultured in the presence of increasing concentrations of Aciclovir (ACV) (**A**), Ganciclovir (GCV) (**B**), Clofarabine (CFB) (**C**), Stavudine (d4T) (**D**), Didanosine (ddI) (**E**) and Abacavir (ABC) (**F**). Cells were incubated with HIV-1-GFP and the percentage of infected GFP^+^ cells was measured by flow cytometry. Cells expressing SAMHD1 (Vpx^−^) (blue filled circles), cells transduced with Vpx (Vpx^+^) (red filled circles) are shown. Infectivity data is plotted as the normalised percentage of GFP^+^ cells against drug concentrations. Error bars represent the standard deviation from at least 2 independent experiments.

No differences were observed in the anti-HIV-1 activities of ACV and GCV in PMA^−^ THP-1 cells upon Vpx transduction (Table 4). By contrast, in PMA^+^ cells, transduction with Vpx reduced ACV anti-HIV-1 activity 27-fold (*p* = 0.0004) (Fig. 4A) and GCV anti-HIV-1 activity was entirely lost (EC_50_ > 200 μM), (*p* < 0.0001) (Fig. 4B). The anti-HIV-1 activity of CFB decreased in both PMA^−^ and PMA^+^ THP-1 cells upon Vpx expression (Table 4). However, in the PMA^−^ cells the EC_50_ was still greater than the observed CFB CC_50_ (Supplementary Table S2). In PMA^+^ cells, introduction of Vpx reduced CFB anti-HIV-1 activity 4-fold (Fig. 4C). This maintained the EC_50_ at lower than cytotoxic levels but the reduction was significantly lower than that observed for ACV and GCV. Nevertheless, the data show that removal of SAMHD1 activity in differentiated THP-1 cells by Vpx-mediated proteasomal degradation results in reduction of anti-HIV-1 activity of these anti-herpes and anti-cancer agents.

Evaluation of the NRTIs showed that Vpx transduction of THP-1 PMA^−^ cells resulted in only small effects on the anti-HIV-1 activities of d4T and ddI (2–3 fold lower) and no difference with ABC (Table 4). By contrast, in THP-1 PMA^+^ cells, the anti-HIV-1 activities of all NRTIs decreased significantly when cells were transduced with Vpx, d4T 35-fold, ddI 55-fold and ABC 187-fold (Fig. 4D–F). By comparison, Vpx transduction of PMA^−^ or PMA^+^ THP-1 cells had no effect on anti-HIV-1 activities of control NVP and RAL (Table 4) suggesting that the decreased anti-HIV-1 activities of the NRTIs is a result of competition from an increased cellular dNTP pool following removal of SAMHD1.

## Discussion

This study was initiated to identify nucleotide analogue drugs that would activate or inhibit SAMHD1 to facilitate an improved understanding of the effect of SAMHD1 on HIV-1 replication. To this end, we had two alternative but not mutually exclusive hypotheses. First that a drug affecting SAMHD1 activity, either positively or negatively, would impact dNTP pool sizes thereby affecting HIV-1 reverse transcription. Second, that cell dependent changes in SAMHD1 activity and the resulting changes in dNTP levels might influence the effects of the drugs themselves on HIV-1 replication. The data obtained and discussed below, provided clear evidence supporting the second hypothesis, at least in the majority of cases. This would suggest that altering SAMHD1 activity might provide a means for enhancing drug effectiveness. Surprisingly our data also provided unambiguous evidence for direct effects of ACV, GCV and CFB not normally thought of as antiretroviral drugs, on HIV-1 replication in certain non-proliferating cell types.

### Control of dNTP pools in cycling and non-cycling cells

Cellular dNTP concentrations vary by a factor of at least 100 between rapidly proliferating cells typified by activated lymphocytes or tumour cells, where they are required by DNA polymerases to actively replicate the genome, and non-proliferating, terminally-differentiated cells such as monocyte-derived macrophages where they are important for DNA repair and mitochondrial DNA synthesis^52^,^53^,^54^. These levels are set by controlling the activity of the enzymes ribonucleotide reductase (RNR), crucial for dNTP biosynthesis, and SAMHD1, that catalyses dNTP degradation^55^. Regulation of RNR and SAMHD1 activity is provided by a range of mechanisms including synthesis, degradation, allostery and phosphorylation^56^,^57^,^58^ that allow control of dNTPs at levels ranging from 20 nM to 2 μM^32^. Given the requirement for dNTPs in HIV-1 replication, such a range of concentrations might be expected to have profound effects both on the apparent efficiency of HIV-1 reverse transcription in different cell types as well as on interactions between RT and NRTIs.

### *In vitro* measurements of SAMHD1 activity

It is possible that any concentration dependent drug effects on SAMHD1 activity might complicate interpretation of cell-based assays to examine nucleoside analogue effects on HIV-1 replication. Our *in vitro* experiments, summarized in Table 1 now allow us to exclude this possibility in the majority of cases. No evidence for SAMHD1 inhibition was seen with any analogue tested. Similarly GCV-TP, d4T-TP, ABC-TP and CBV-TP showed no activity as activator or substrate, implying no direct effects on SAMHD1. By contrast both ACV-TP and ddI-TP were allosteric activators of SAMHD1 though ddI-TP was much less efficient than the natural activator GTP. Thus for these two compounds a role for activation cannot formally be excluded. However, ddI and ddI-MP are poor substrates for further phosphorylation by cellular kinases^59^ and ddI is rapidly metabolized to ddA-TP in cells^49^ making a direct effect of ddI-TP on SAMHD1 activity unlikely.

### Efficacy of NRTIs in cycling and non-cycling cells

NRTIs compete with endogenous dNTPs for incorporation into newly reverse transcribed HIV-1 DNA and act as chain terminators. Therefore, given equal cellular uptake and comparable conversion efficiency to triphosphorylated forms, their efficacy would be predicted to depend on the level of competing natural dNTPs. Thus in non-dividing cells further depletion of the dNTP pool by catalytically active SAMHD1 might be expected to profoundly reduce the doses of NRTIs required to inhibit HIV-1 infectivity. This notion is supported by our observations of increased NRTI efficacy in PMA-differentiated U937 cells transduced with active SAMHD1 (Table 3) and deceased NRTI efficacy in THP-1 cells were SAMHD1 has been targeted for degradation through expression of Vpx (Table 4). In addition, although minor, the presence of SAMHD1 in proliferating myeloid cells results in enhancement of d4T and ddI anti-HIV-1 activity (Tables 3 and 4).

### Anti HIV-1 activity of CFB

Evaluation of our data also showed that SAMHD1 could enhance the anti-HIV-1 activity of the anticancer agent CFB (Tables 3 and 4). The anticancer agents 5-azacytidine, CFB, and resveratrol have previously been shown to exhibit potent anti-HIV-2 activity with EC_50_ values for 5-azacytidine, clofarabine and resveratrol being significantly lower for HIV-2 compared to HIV-1^60^. More recently CFB was shown to have significant anti-HIV-1 activity in primary macrophages acting both to reduce dNTP concentrations by inhibiting RNR and HIV-1 viral DNA via RT^61^. Our data support these previous observations and also that concentration of CFB for full anti-HIV-1 activity was significantly decreased upon differentiation of monocytes (Table 2). Moreover, CFB anti-HIV-1 activity was enhanced 22-fold in the presence of SAMHD1 in PMA-differentiated monocytes (Table 3), consistent with a mechanism involving chain termination of HIV-1 DNA synthesis in a manner analogous to NRTIs.

### Anti HIV-1 activity of ACV and GCV

ACV and GCV are normally considered as herpes virus specific drugs. Their incorporation into growing DNA results in chain termination; selectivity is determined by the viral thymidine kinase, required for the efficient conversion into the mono and subsequent triphosphorylated derivatives needed for incorporation into DNA^62^. However they have also been shown to reduce HIV-1 load and/or delay disease progression^63^,^64^,^65^ with ACV acting as a chain terminator of reverse transcriptase^66^. If so, how does phosphorylation occur in HIV-1 infected individuals? One school of thought postulates that kinases from co-infecting human herpesviruses are involved^67^,^68^. Against this idea are studies that show that herpesvirus co-infection is not required for ACV activity against HIV-1^69^ implying that as yet uncharacterised human kinases must be involved^70^.

Our data now show that SAMHD1 has a pronounced effect on the anti-HIV-1 activity of the drugs ACV and GCV when tested in myeloid derived cell lines. ACV and GCV do not display substantive anti-HIV-1 activities in proliferating monocytes (Table 2). However, in differentiated cells expressing SAMHD1, where the dNTP pool levels are much lower^52^,^53^,^71^ ACV and GCV anti-HIV-1 activities were greatly enhanced (Figs 2 and 3) and this enhancement could be abrogated by introduction of Vpx to reduce SAMHD1 triphosphohydrolase activity (Fig. 4). These data indicate that the anti-herpes virus drugs are most active in non-proliferating cells with low dNTP levels and are fully consistent with the observation that the anti-HIV-1 activity of ACV can be potentiated by ribavirin, a drug that depletes intracellular dGTP pools^68^ and that ACV can inhibit HIV-1 replication in resting T-cells^69^. Our observations also support the idea that a cellular kinase, expressed in THP-1 and U937 cells, is responsible for initial phosphorylation. Such cells might provide starting material for the purification and further characterization of this enzyme.

In light of these results, the potential utility of nonstandard antiviral and anticancer drugs to target HIV-1 in differentiated cells low in dNTPs might also be considered. For example, our data, coupled with the low cost and favourable safety profile^72^ of ACV prompt the question of whether ACV might find a niche role in the treatment or prevention of HIV-1. Thus, it is tempting to speculate that ACV might be useful for the prevention of mother to child transmission, or for reducing reservoir size, in resource poor settings where viral load suppression of the expectant mother may be less than complete and treatment of the new-born infant could be initiated before results of tests for transmission become available. We are currently testing this idea in a variety of primary culture systems with low dNTP concentrations such monocyte-derived macrophages or resting T-cells, aiming to identify important HIV-1 target cells that are susceptible to ACV treatment.

## Methods

### Synthesis of abacavir-5′-triphosphate and carbovir-5′-triphosphate

The synthesis of the ammonium salt of abacavir-5′-triphosphate (ABC-TP) was performed as described^73^ with minor modifications. Under a nitrogen atmosphere 364 mg (1.27 mmol) of Abacavir was dissolved in 9.5 mL anhydrous pyridine and cooled to −35 °C. Then 1.65 mL (1.65 mmol, 1.3 eq) of a 1 M solution of 5-chloro-*cyclo*Saligenyl-phosphorchloridate in THF was added portion-wise over a period of 4 h. The reaction mixture was slowly warmed to room temperature and all volatiles were evaporated. The residue was co-evaporated three times with toluene (3 × 5 mL) and finally once with CH_2_Cl_2_. The crude product was purified by flash column chromatography (CH_2_Cl_2_/CH_3_OH + 1% of acetic acid, 95:5 to 90:10) to give 490 mg of the *cyclo*Saligenyl-triester with minor impurities. Then 47.5 mg (97.2 μmol) of this phosphate triester were dissolved in 1 mL anhydrous DMF. A solution of 96.3 mg (146 μmol) bis-tetrabutylammonium hydrogen pyrophosphate in 0.7 mL DMF, which had been stirred for 1 h over 4 Å molecular sieves, was added in one aliquot. After 72 h the solvent was removed under reduced pressure and the residue was purified by reverse phase flash column chromatography (H_2_O/CH_3_CN, 99:1 to 0:100), ion exchange (NH_4_^+^) on Dowex (50WX8) and again reversed phase flash column chromatography (H_2_O/CH_3_CN, 100:1 to 0:100) to give 20.5 mg (34.5 μmol) of the triphosphate as a colourless solid after freeze drying. ^**1**^**H NMR:** (600 MHz, D_2_O): *δ* [ppm] = 7.99 (s, 1H), 6.19–6.26 (m, 1H), 5.93–5.89 (m, 1H), 5.52–5.48 (m, 1H), 4.10–4.06 (m, 1 H), 4.03–3.98 (m, 1H), 3.23–3.17 (m, 1H), 2.90–2.76 (m, 2H), 1.78–1.71 (m, 1H), 0.98–0.93 (m, 2H), 0.76–0.72 (m, 2H). ^**13**^**C NMR:** (151 MHz, D_2_O): *δ* [ppm] = 148.9, 139.3, 138.5, 129.2, 68.3, 59.9, 45.5, 33.6, 22.9, 6.7, 6.7. ^**31**^**P NMR:** (243 MHz, D_2_O): −10.73, −10.91, −23.27. **HRMS** (ESI^−^, m/z): calcd. for C_14_H_21_N_6_O_10_P_3_: 525.0459 [M-H]^−^, found 525.0360 [M-H]^−^. **IR (ATR):** wavenumber [cm^−1^] = 2988, 2884, 1644, 1445, 1407, 1210, 1118, 891, 832, 484.

### Synthesis of abacavir-5′-triphosphate and carbovir-5′-triphosphate

Carbovir 5′-triphosphate (CBV-TP) was prepared similarly. Amounts used were: 308 mg (1.25 mmol) carbovir, 9.5 mL anhydrous pyridine and 1.62 mL (1.62 mmol, 1.3 eq) of 1 M 5-chloro-*cyclo*Saligenyl-phosphorcloridate solution (THF). The crude product was purified by flash column chromatography (CH_2_Cl_2_/CH_3_OH + 1% of acetic acid, 95:5 to 90:10) to give 219 mg of the *cyclo*Saligenyl-triester with minor impurities. In the second step 47.7 mg (106 μmol) *cyclo*Sal-triester of carbovir in 1 mL dry DMF and 105 mg (159 μmol) of bis-tetrabutylammonium hydrogen pyrophosphate in 0.8 mL DMF were reacted. After purification and freeze-drying 12.3 mg (22.1 μmol) of the triphosphate was obtained as a colorless solid. ^**1**^**H NMR:** (600 MHz, D_2_O): *δ* [ppm] = 8.00 (s, 1H), 6.29–6.26 (m, 1H), 5.94–5.92 (m, 1H), 5.54–5.49 (m, 1H), 4.08–3.98 (m, 2 H), 3.23–3.17 (m, 1H), 2.85–2.77 (m, 1H), 1.78–1.71 (m, 1H). ^**13**^**C NMR:** (151 MHz, D_2_O): *δ* [ppm] = 158.6, 153.7, 151.0, 138.4, 138.3, 129.3, 115.2, 68.6, 60.1, 45.4, 33.8. ^**31**^**P NMR:** (243 MHz, D_2_O): −10.87, −10.98, −23.31. **HRMS** (ESI^−^, m/z): calcd. for C_11_H_16_N_5_O_11_P_3_: 485.9986 [M-H]^−^, found 486.0020 [M-H]^−^. **IR (ATR):** wavenumber [cm^−1^] = 2988, 1686, 1610 1407, 1214, 1056, 1004, 948, 893, 473.

### Measurement of *in vitro* dNTP triphosphohydrolase activity

SAMHD1 hydrolysis, activation and inhibition by nucleotide analogues was quantified using a continuous coupled assay employing the fluorescent phosphate biosensor MDCC-PBP to measure phosphate release from combined SAMHD1 triphosphohydrolase and *S. cerevisiae* Ppx1 exopolyphosphatase activity, described previously^21^. SAMHD1 and Ppx1 were expressed as strep-tagged fusion proteins and purified as described previously^19^,^21^. Didanosine triphosphate (ddI-TP) and dideoxy ATP (ddATP) were from Trilink Biotechnologies, Stavudine triphosphate (d4T-TP) was from Jena biosciences and Chemcyte. Abacavir triphosphate and Carbovir triphosphate were chemically synthesised in house from abacavir and carbovir as described above. Experiments were performed in 20 μL reaction volumes in 384-well low-protein-binding microplates (Corning, USA). In typical experiments, solutions containing SAMHD1, Ppx1, MDCC-PBP and activator (GTP, ddI-TP or d4T-TP), or activator plus inhibitor, were incubated for 5 min in assay buffer (20 mM Tris pH 8.0, 150 mM NaCl, 5 mM MgCl_2_ and 2 mM TCEP) at 25 °C before the reaction was initiated by the addition of substrate (TTP, ddI-TP or d4T-TP). The final concentrations were 100 nM SAMHD1(115–626), 10 nM Ppx1, 2.5 μM BSA, 40 μM MDCC-PBP (80 μM in ABC-TP and CBV-TP assays). Activator, substrate and inhibitor were added at concentrations described below. The fluorescence intensity increase resulting from the phosphate-product binding to MDCC-PBP was recorded at 430 nm excitation and 465 nm emission over 10–30 min in a Clariostar multiwell plate reader (BMG). Steady-state rates were obtained from the fluorescence time courses by fitting a linear function with maximal slope using the Mars software (BMG). Apparent dissociation constant of ddI-TP binding (*K*_*S*_), catalytic constant (*k*_*cat*_), maximal rate (*k*_*max*_) and the ddI-TP concentration at half maximal activation (*K*_*a*_) were determined by nonlinear least squares fitting using a Hill-function in the software package Grafit 7.0.3 (Erithacus software, UK). All measurements were performed at least in triplicate and data are represented as mean ± SEM.

### Cell lines and reagents

Cell lines were obtained from the Programme EVA Centre for AIDS Reagents and tested free for mycoplasma contamination. Jurkat^74^, H9^75^, U937^76^ and THP-1 cells^77^ were maintained in RPMI-1640 medium containing [L]-glutamine (Life Technologies), supplemented with heat-inactivated 10% foetal bovine serum (Labtech), 100 U penicillin-G/mL and 10 μg streptomycin/mL. 293T cells were maintained in DMEM medium containing [D]-glucose, [L]-glutamine and sodium pyruvate (Life Technologies), supplemented with 10% foetal bovine serum (Labtech), 100 U penicillin-G/mL and 10 μg streptomycin/mL. ACV and GCV were from Sequoia Research products (UK), CFB from Sigma-Aldrich (USA), d4T from Santa Cruz Biotechnology (USA), ddI and ABC from Stratech Scientific (UK), and Nevirapine (NVP) and Raltegravir (RAL) from Cayman Chemical (USA). All compounds were prepared at 100 mM in dimethyl sulphoxide and stored at −20 °C until use.

### Virus production

SAMHD1 and HIV-2 Vpx DNA sequences were inserted into pLGatewayIeYFP^78^. The active site mutation HD206-7AA was created by PCR-based site-directed mutagenesis (QuickChange-II XL, Agilent). Virus-like particles (VLPs) were produced by co-transfection of pVSV-G^79^, pKB4^80^ and the pLGatewayIeYFP plasmids (SAMHD1, HD206-7AA or Vpx) into 293T cells and harvesting 48 h after transfection. HIV-1-GFP was made by co-transfection of pVSV-G, p8.91^81^ and pCSGW-GFP^82^. Viruses were titrated in U937 cells and stocks stored at −80 °C until use.

### Cytotoxicity assays

H9, Jurkat and undifferentiated U937 and THP-1 (PMA^−^) cells (6 × 10^5^ cells/mL) were treated with increasing concentrations of nucleoside analogues (0–200 μM; except CFB, 0–1.6 μM) and incubated at 37 °C in 5% CO_2_ for 48 h. Medium was then replaced and cells were further incubated for 72 h. Non-dividing U937 and THP-1 (PMA^+^) cells (6 × 10^5^ cells/mL) were differentiated by addition of 100 nM phorbol-12-myristate-13-acetate (PMA^+^) for 48 h and then treated with increasing concentrations of nucleoside analogues as described above. The percentage of live cells was determined by flow cytometry using Live/Dead fixable blue-fluorescent dyes (Invitrogen) and a Fortessa X20 analyser (BD Biosciences). Data were analysed using the FlowJo software suite.

### Antiviral assays

H9 and Jurkat cells (6 × 10^5^ cells/mL) were treated with increasing concentrations of nucleoside analogues (0–200 μM; except CFB, 0–1.6 μM) and incubated for 4 h at 37 °C in 5% CO_2_. Cells were then infected with HIV-1-GFP by spinoculation at 800 × *g* for 90 min in the presence of 1 μg/mL polybrene, and further incubated at 37 °C in 5% CO_2_ for 72 h. Undifferentiated U937 and THP-1 (PMA^−^) cells (6 × 10^5^ cells/mL) were treated with increasing concentrations of nucleoside analogues (0–200 μM; except CFB, 0–1.6 μM) and incubated at 37 °C in 5% CO_2_ for 48 h. Cells were then infected with HIV-1-GFP by spinoculation at 800 × *g* for 90 min in the presence of 1 μg/mL polybrene, and further incubated at 37 °C in 5% CO_2_ for 72 h. Non-dividing U937 and THP-1 (PMA^+^) cells (6 × 10^5^ cells/mL) were differentiated by addition of 100 nM PMA (PMA^+^) for 48 h and treated with increasing concentrations of nucleoside analogues as described above. Cells were then infected with HIV-1-GFP in the presence of 1 μg/mL polybrene and further incubated at 37 °C in 5% CO_2_ for 72 h. The percentage of GFP^+^cells was determined by one-colour flow cytometry using a Fortessa X20 analyser (BD Biosciences) and the data analysed using the FlowJo software suite.

Antiviral assays were further performed in monocytoid cells transduced with YFP-expressing VLPs containing WT SAMHD1, HD206-7AA or Vpx. Briefly, U937 cells (6 × 10^5^ cells/mL) were transduced with WT SAMHD1-YFP or HD206-7AA-YFP VLPs by spinoculation at 800 × *g* for 90 min in the presence of 1 μg/mL polybrene. Similarly, THP-1 cells (6 × 10^5^ cells/mL) were transduced with Vpx-YFP VLPs by spinoculation at 800 × *g* for 90 min in the presence of 1 μg/mL polybrene. Cells were incubated at 37 °C in 5% CO_2_ and after 72 h, PMA^+^ cells (6 × 10^5^ cells/mL) were treated with 100 nM PMA and PMA^−^ cells (7.5 × 10^4^ cells/mL) were left untreated. Cells were then incubated at 37 °C in 5% CO_2_ for 48 h. Nucleoside analogues were added at increasing concentrations (0–200 μM; except CFB, 0–1.6 μM) and cells were further incubated for 48 h. PMA^−^ cells were then infected with HIV-1-GFP by spinoculation at 800 × *g* for 90 min in the presence of 1 μg/mL polybrene, PMA^+^ cells were infected with HIV-1-GFP in the presence of 1 μg/mL polybrene only. Cells were incubated for 72 h at 37 °C in 5% CO_2_ and then analysed by two-colour flow cytometry using a Fortessa X20 analyser (BD Biosciences). Data were analysed using the FlowJo software suite.

### Statistical analysis

The cytotoxic concentration of nucleoside analogue that inhibits cell viability by 50% (CC_50_) and the effective concentration of nucleoside analogue that inhibits infection of cells by 50% (EC_50_) were calculated for each independent experiment by non-linear regression using GraphPad Prism v6.00 for Windows (GraphPad Software, San Diego, CA). Non-ambiguous values were subjected to a replicate test to calculate confidence intervals of 95% (95% CI). CC_50_ and EC_50_ values were determined for at least two independent experiments for each nucleoside analogue and a selectivity index (SI) also calculated, (SI = CC_50_/EC_50_). EC_50_ values for each replicate were used to perform an unpaired two-tailed t-test to determine significant differences between U937 cells expressing SAMHD1 *wt* or HD206-7AA mutant and THP-1 cells expressing Vpx or untransduced cells. P values of <0.005 were considered statistically significant.

## Additional Information

**How to cite this article:** Ordonez, P. *et al*. SAMHD1 enhances nucleoside-analogue efficacy against HIV-1 in myeloid cells. *Sci. Rep.* **7**, 42824; doi: 10.1038/srep42824 (2017).

**Publisher's note:** Springer Nature remains neutral with regard to jurisdictional claims in published maps and institutional affiliations.

## Supplementary Material

Supplementary Material

## Figures and Tables

**Table 1:** Nucleoside analogues selected for this study.

Nucleoside analogue^a^	Generic name	Therapeutic use^b^	Base	References
*Antiherpesvirus*				
ACV	Aciclovir	HSV-1, HSV-2 and VSV treatment, EBV and CMV prevention	G	(37, 38)
GCV	Ganciclovir	Treatment of CMV infections	G	(39)
*Anticancer*				
CFB	Clofarabine	Treatment of ALL in children after failure.	A^c^	(40, 41)
*NRTIs*				
D4T	Stavudine	HIV/AIDS therapy	T	(42, 43)
ddI	Didanosine	HIV/AIDS therapy	H^d^	(44, 48, 49)
ddA^e^	Dideoxy A		A	
ABC	Abacavir	HIV/AIDS therapy	Φ^f^	(45, 50, 51)
CBV^g^	Carbovir		G	

^a^**ACV**: Acycloguanosine; **GCV**: 2′-Nor-2′-deoxyguanosine; **CFB**: 2-chloro-9-(2′-Deoxy-2′-fluoro-beta-D-arabinofuranosyl)adenine; d4T: 2′,3′-didehydro-2′,3′-dideoxythymidine; **ddI**: 2′,3′-dideoxyinosine; **ddA**: 2,3′-Dideoxyadenosine; **ABC**: (1S, 4R)-4-[2-amino-6-(cyclopropylamino)-9H-purin-9-yl]cyclopent-2-en-1-yl}methanol; **CBV** (1S, 4R)-4-[2-amino-6-oxo-1,6-dihydro-9H-purin-9-yl]cyclopent-2-en-1-yl]methanol.

^b^HSV-1: Herpes simplex virus type I; HSV-2: Herpes simplex virus type II; VSV: Varicella zoster virus; EBV: Epstein-Barr virus; CMV: Cytomegalovirus; ALL: Acute Lymphoblastic Leukaemia.

^c^2-Chloro-adenine.

^d^Hypoxanthine.

^e^Cellular converted form of ddI.

^f^Non-natural (2-amino-6-(cyclopropylamino)purine).

^g^Cellular converted active form of ABC.

**Table 2:** Anti-HIV-1 activity of nucleoside analogues in monocytoid cell lines.

*Nucleoside analogue*		EC_50_ (μM)^a^ [95% CI]^b^		
	U937		THP1	
	PMA^−^	PMA^+^	PMA^−^	PMA^+^
ACV	>200	52.2 [49.5–54.9]	>200	0.657 [0.476–0.838]
GCV	>200	>200	>200	0.367 [0.322–0.412]
CFB	0.119 [0.101–0.137]	0.037 [0.012–0.061]	0.132 [0.055–0.210]	0.003 [0.002–0.004]
d4T	6.55 [1.83–11.3]	7.04 [6.97–7.11]	6.21 [5.91–6.52]	3.95 [3.05–4.85]
ddI	45.8 [29.2–62.5]	2.45 [0.563–4.34]	2.03 [1.74–2.32]	0.093 [0.065–0.121]
ABC	14.1 [5.07–23.0]	7.47 [6.67–8.27]	0.493 [0.142–0.844]	0.031 [0.022–0.040]
NVP	0.505 [0.126–0.883]	0.118 [0.064–0.171]	0.369 [0.232–0.506]	0.219 [0.201–0.237]
RAL	0.013 [0.012–0.014]	0.063 [0.038–0.088]	0.044 [0.035–0.053]	0.037 [0.021–0.046]

^a^EC_50_ is the effective concentration of drug that inhibits infection of cells by 50% measured as percentage of GFP^+^ cells. Values were calculated by non-linear regression for at east two independent experiments.

^b^95% confidence interval. Non-ambiguous values were subject to a replicate test.

**Table 3:** Anti-HIV-1 activity of nucleoside analogues in SAMHD1 expressing U937 cells.

*Nucleoside analogue*	PMA^−^				PMA^+^			
	EC_50_ (μM)^a^ [95% CI]^b^		Fold	p^c^	EC_50_ (μM) [95% CI]		Fold	p
	SAMHD1	HD206/7AA			SAMHD1	HD206/7AA		
ACV	>200	>200	—	—	0.652 [0.175–1.13]	50.9 [46.1–55.8]	78	<0.0001
GCV	>200	>200	—	—	0.876 [0.823–0.929]	>200	>228	<0.0001
CFB	0.138 [0.021–0.255]	0.108 [0.081–0.135]	1	0.0878†	0.0013 [0.000–0.002]	0.029 [0.022–0.036]	22	0.0010
d4T	0.801 [0.531–1.07]	2.71 [1.90–3.52]	3	0.0013	0.528 [0.317–0.739]	5.82 [5.671–5.969]	11	<0.0001
ddI	23.7 [10.6–36.7]	55.1 [44.2–65.9]	2	0.0018	0.109 [0.030–0.188]	8.79 [7.53–10.1]	81	<0.0001
ABC	17.5 [15.9–19.1]	22.1 [7.12–37.1]	1	0.0590†	0.173 [0.164–0.182]	7.06 [4.72–9.40]	41	<0.0007
NVP	0.112 [0.000–0.223]	0.124 [0.000–0.248]	1	0.6442†	0.097 [0.000–0.195]	0.145 [0.113–0.177]	1	0.1297†
RAL	0.011 [0.002–0.020]	0.012 [0.003–0.021]	1	0.2115†	0.070 [0.013–0.127]	0.075 [0.007–0.143]	1	0.7548†

^a^EC_50_ is the effective concentration of drug that inhibits infection of cells by 50% measured as the percentage of GFP^+^ cells in SAMHD1+ or HD206/7AA+ populations. Values were calculated by non-linear regression for at least two independent experiments.

^b^95% confidence interval. Non-ambiguous values were subject to a replicate test.

^c^EC_50_ values for each replicate were used to perform an unpaired two-tailed t-test to determine differences between SAMHD1+ and HD206/7AA+ populations.

^†^Difference not statistically significant.

**Table 4:** Anti-HIV-1 activity of nucleoside analogues in Vpx expressing THP1 cells.

*Nucleoside analogue*	PMA^−^				PMA^+^			
	EC_50_ (μM)^a^ [95% CI]^b^		Fold	p^c^	EC_50_ (μM) [95% CI]		Fold	p
	Vpx^−^	Vpx^+^			Vpx^−^	Vpx^+^		
ACV	>200	>200	—	—	1.08 [0.735–1.43]	29.6 [2.62–56.5]	27	0.0004
GCV	>200	>200	—	—	0.632 [0.596–0.668]	>200	>316	<0.0001
CFB	0.340 [0.118–0.563]	>1.6	>5	<0.0001	0.003 [0.002–0.004]	0.012 [0.003–0.021]	4	0.0017
d4T	4.39 [3.76–5.02]	13.3 [12.1–14.4]	3	0.0001	1.49 [0.501–2.48]	52.2 [33.0–71.5]	35	0.0009
ddI	1.04 [0.863–1.22]	2.73 [1.40–4.07]	3	0.0039	0.079 [0.061–0.096]	4.34 [3.19–5.50]	55	0.0005
ABC	0.459 [0.399–0.519]	0.622 [0.356–0.888]	1	0.0619†	0.038 [0.036–0.040]	7.050 [4.67–9.43]	187	0.0002
NVP	0.244 [0.240–0.248]	0.292 [0.067–0.517]	1	0.1146†	0.223 [0.196–0.250]	0.235 [0.217–0.253]	1	0.0379†
RAL	0.035 [0.017–0.053]	0.043 [0.016–0.070]	1	0.0683†	0.038 [0.020–0.056]	0.037 [0.002–0.072]	1	0.9071†

^a^EC_50_ is the effective concentration of drug that inhibits infection of cells by 50% measured as the percentage of GFP^+^ cells in Vpx^−^ or Vpx^+^ populations. Values were calculated by non-linear regression for at least two independent experiments.

^b^95% confidence interval. Non-ambiguous values were subject to a replicate test.

^c^EC_50_ values for each replicate were used to perform an unpaired two-tailed t-test to determine differences between Vpx^−^ and Vpx^+^ populations.

^†^Difference not statistically significant.

## References

[b1] Hrecka K. . Vpx relieves inhibition of HIV-1 infection of macrophages mediated by the SAMHD1 protein. Nature 474, 658–661, doi: 10.1038/nature10195 (2011). 21720370 PMC3179858

[b2] Laguette N. . SAMHD1 is the dendritic- and myeloid-cell-specific HIV-1 restriction factor counteracted by Vpx. Nature 474, 654–657, doi: 10.1038/nature10117 (2011). 21613998 PMC3595993

[b3] Descours B. . SAMHD1 restricts HIV-1 reverse transcription in quiescent CD4(+) T-cells. Retrovirology 9, 87, doi: 10.1186/1742-4690-9-87 (2012). 23092122 PMC3494655

[b4] Baldauf H. M. . SAMHD1 restricts HIV-1 infection in resting CD4(+) T cells. Nature medicine 18, 1682–1687, doi: 10.1038/nm.2964 (2012). PMC3828732 22972397

[b5] Laguette N. . Evolutionary and functional analyses of the interaction between the myeloid restriction factor SAMHD1 and the lentiviral Vpx protein. Cell host & microbe 11, 205–217, doi: 10.1016/j.chom.2012.01.007 (2012). 22305291 PMC3595996

[b6] Lim E. S. . The ability of primate lentiviruses to degrade the monocyte restriction factor SAMHD1 preceded the birth of the viral accessory protein Vpx. Cell host & microbe 11, 194–204, doi: 10.1016/j.chom.2012.01.004 (2012). 22284954 PMC3288607

[b7] Lahouassa H. . SAMHD1 restricts the replication of human immunodeficiency virus type 1 by depleting the intracellular pool of deoxynucleoside triphosphates. Nature immunology 13, 223–228, doi: 10.1038/ni.2236 (2012). 22327569 PMC3771401

[b8] St Gelais C. . SAMHD1 restricts HIV-1 infection in dendritic cells (DCs) by dNTP depletion, but its expression in DCs and primary CD4^+^ T-lymphocytes cannot be upregulated by interferons. Retrovirology 9, 105, doi: 10.1186/1742-4690-9-105 (2012). 23231760 PMC3527137

[b9] Kim B., Nguyen L. A., Daddacha W. & Hollenbaugh J. A. Tight interplay among SAMHD1 protein level, cellular dNTP levels, and HIV-1 proviral DNA synthesis kinetics in human primary monocyte-derived macrophages. The Journal of biological chemistry 287, 21570–21574, doi: 10.1074/jbc.C112.374843 (2012). 22589553 PMC3381122

[b10] Goldstone D. C. . HIV-1 restriction factor SAMHD1 is a deoxynucleoside triphosphate triphosphohydrolase. Nature 480, 379–382, doi: 10.1038/nature10623 (2011). 22056990

[b11] Beloglazova N. . Nuclease activity of the human SAMHD1 protein implicated in the Aicardi-Goutieres syndrome and HIV-1 restriction. The Journal of biological chemistry 288, 8101–8110, doi: 10.1074/jbc.M112.431148 (2013). 23364794 PMC3605629

[b12] Choi J., Ryoo J., Oh C., Hwang S. & Ahn K. SAMHD1 specifically restricts retroviruses through its RNase activity. Retrovirology 12, 46, doi: 10.1186/s12977-015-0174-4 (2015). 26032178 PMC4450836

[b13] Ryoo J. . The ribonuclease activity of SAMHD1 is required for HIV-1 restriction. Nature medicine 20, 936–941, doi: 10.1038/nm.3626 (2014). PMC4318684 25038827

[b14] Seamon K. J., Sun Z., Shlyakhtenko L. S., Lyubchenko Y. L. & Stivers J. T. SAMHD1 is a single-stranded nucleic acid binding protein with no active site-associated nuclease activity. Nucleic acids research 43, 6486–6499, doi: 10.1093/nar/gkv633 (2015). 26101257 PMC4513882

[b15] Goncalves A. . SAMHD1 is a nucleic-acid binding protein that is mislocalized due to aicardi-goutieres syndrome-associated mutations. Human mutation 33, 1116–1122, doi: 10.1002/humu.22087 (2012). 22461318

[b16] Ji X., Tang C., Zhao Q., Wang W. & Xiong Y. Structural basis of cellular dNTP regulation by SAMHD1. Proceedings of the National Academy of Sciences of the United States of America 111, E4305–4314, doi: 10.1073/pnas.1412289111 (2014). 25267621 PMC4205617

[b17] Ji X. . Mechanism of allosteric activation of SAMHD1 by dGTP. Nature structural & molecular biology 20, 1304–1309, doi: 10.1038/nsmb.2692 (2013). PMC3833828 24141705

[b18] Zhu C. . Structural insight into dGTP-dependent activation of tetrameric SAMHD1 deoxynucleoside triphosphate triphosphohydrolase. Nat Commun 4, 2722, doi: 10.1038/ncomms3722 (2013). 24217394

[b19] Arnold L. H. . Phospho-dependent Regulation of SAMHD1 Oligomerisation Couples Catalysis and Restriction. PLoS pathogens 11, e1005194, doi: 10.1371/journal.ppat.1005194 (2015). 26431200 PMC4592219

[b20] Hansen E. C., Seamon K. J., Cravens S. L. & Stivers J. T. GTP activator and dNTP substrates of HIV-1 restriction factor SAMHD1 generate a long-lived activated state. Proceedings of the National Academy of Sciences of the United States of America 111, E1843–1851, doi: 10.1073/pnas.1401706111 (2014). 24753578 PMC4020072

[b21] Arnold L. H., Kunzelmann S., Webb M. R. & Taylor I. A. A continuous enzyme-coupled assay for triphosphohydrolase activity of HIV-1 restriction factor SAMHD1. Antimicrobial agents and chemotherapy 59, 186–192, doi: 10.1128/AAC.03903-14 (2015). 25331707 PMC4291348

[b22] Yan J. . Tetramerization of SAMHD1 is required for biological activity and inhibition of HIV infection. The Journal of biological chemistry 288, 10406–10417, doi: 10.1074/jbc.M112.443796 (2013). 23426366 PMC3624423

[b23] Welbourn S., Dutta S. M., Semmes O. J. & Strebel K. Restriction of virus infection but not catalytic dNTPase activity is regulated by phosphorylation of SAMHD1. Journal of virology 87, 11516–11524, doi: 10.1128/JVI.01642-13 (2013). 23966382 PMC3807338

[b24] Cribier A., Descours B., Valadao A. L., Laguette N. & Benkirane M. Phosphorylation of SAMHD1 by cyclin A2/CDK1 regulates its restriction activity toward HIV-1. Cell Rep 3, 1036–1043, doi: 10.1016/j.celrep.2013.03.017 (2013). 23602554

[b25] White T. E. . The retroviral restriction ability of SAMHD1, but not its deoxynucleotide triphosphohydrolase activity, is regulated by phosphorylation. Cell host & microbe 13, 441–451, doi: 10.1016/j.chom.2013.03.005 (2013). 23601106 PMC3864637

[b26] Pauls E. . Cell cycle control and HIV-1 susceptibility are linked by CDK6-dependent CDK2 phosphorylation of SAMHD1 in myeloid and lymphoid cells. Journal of immunology 193, 1988–1997, doi: 10.4049/jimmunol.1400873 (2014). 25015816

[b27] St Gelais C. . Identification of cellular proteins interacting with the retroviral restriction factor SAMHD1. Journal of virology 88, 5834–5844, doi: 10.1128/JVI.00155-14 (2014). 24623419 PMC4019113

[b28] Ruiz A. . Cyclin D3-dependent control of the dNTP pool and HIV-1 replication in human macrophages. Cell cycle 14, 1657–1665, doi: 10.1080/15384101.2015.1030558 (2015). 25927932 PMC4614030

[b29] Pauls E. . Palbociclib, a selective inhibitor of cyclin-dependent kinase4/6, blocks HIV-1 reverse transcription through the control of sterile alpha motif and HD domain-containing protein-1 (SAMHD1) activity. AIDS 28, 2213–2222, doi: 10.1097/QAD.0000000000000399 (2014). 25036183

[b30] Kyei G. B., Cheng X., Ramani R. & Ratner L. Cyclin L2 is a critical HIV dependency factor in macrophages that controls SAMHD1 abundance. Cell host & microbe 17, 98–106, doi: 10.1016/j.chom.2014.11.009 (2015). 25532805 PMC4297224

[b31] Badia R. . The G1/S Specific Cyclin D2 Is a Regulator of HIV-1 Restriction in Non-proliferating Cells. PLoS pathogens 12, e1005829, doi: 10.1371/journal.ppat.1005829 (2016). 27541004 PMC4991798

[b32] Amie S. M., Noble E. & Kim B. Intracellular nucleotide levels and the control of retroviral infections. Virology 436, 247–254, doi: 10.1016/j.virol.2012.11.010 (2013). 23260109 PMC3804251

[b33] O’Brien W. A. . Kinetics of human immunodeficiency virus type 1 reverse transcription in blood mononuclear phagocytes are slowed by limitations of nucleotide precursors. Journal of virology 68, 1258–1263 (1994). 7507180 10.1128/jvi.68.2.1258-1263.1994 PMC236573

[b34] Amie S. M. . Anti-HIV host factor SAMHD1 regulates viral sensitivity to nucleoside reverse transcriptase inhibitors via modulation of cellular deoxyribonucleoside triphosphate (dNTP) levels. The Journal of biological chemistry 288, 20683–20691, doi: 10.1074/jbc.M113.472159 (2013). 23744077 PMC3711331

[b35] Huber A. D. . SAMHD1 has differential impact on the efficacies of HIV nucleoside reverse transcriptase inhibitors. Antimicrobial agents and chemotherapy 58, 4915–4919, doi: 10.1128/AAC.02745-14 (2014). 24867973 PMC4136039

[b36] Ballana E. . SAMHD1 specifically affects the antiviral potency of thymidine analog HIV reverse transcriptase inhibitors. Antimicrobial agents and chemotherapy 58, 4804–4813, doi: 10.1128/AAC.03145-14 (2014). 24913159 PMC4136047

[b37] Elion G. B. The biochemistry and mechanism of action of acyclovir. The Journal of antimicrobial chemotherapy 12 Suppl B, 9–17 (1983). 10.1093/jac/12.suppl_b.9 6313600

[b38] Eizuru Y. Development of new antivirals for herpesviruses. Antiviral chemistry & chemotherapy 14, 299–308 (2003). 14968936 10.1177/095632020301400602

[b39] Crumpacker C. S. Ganciclovir. The New England journal of medicine 335, 721–729, doi: 10.1056/NEJM199609053351007 (1996). 8786764

[b40] Bonate P. L. . Discovery and development of clofarabine: a nucleoside analogue for treating cancer. Nature reviews. Drug discovery 5, 855–863, doi: 10.1038/nrd2055 (2006). 17016426

[b41] Xie K. C. & Plunkett W. Deoxynucleotide pool depletion and sustained inhibition of ribonucleotide reductase and DNA synthesis after treatment of human lymphoblastoid cells with 2-chloro-9-(2-deoxy-2-fluoro-beta-D-arabinofuranosyl) adenine. Cancer research 56, 3030–3037 (1996). 8674058

[b42] Baba M. . Both 2′,3′-dideoxythymidine and its 2′,3′-unsaturated derivative (2′,3′-dideoxythymidinene) are potent and selective inhibitors of human immunodeficiency virus replication *in vitro*. Biochem Bioph Res Co 142, 128–134 (1987). 10.1016/0006-291x(87)90460-8 3028398

[b43] Lin T. S., Schinazi R. F. & Prusoff W. H. Potent and selective *in vitro* activity of 3′-deoxythymidin-2′-ene (3′-deoxy-2′,3′-didehydrothymidine) against human immunodeficiency virus. Biochemical pharmacology 36, 2713–2718 (1987). 2443141 10.1016/0006-2952(87)90253-x

[b44] Ahluwalia G. . Initial studies on the cellular pharmacology of 2′,3′-dideoxyinosine, an inhibitor of HIV infectivity. Biochemical pharmacology 36, 3797–3800 (1987). 3120727 10.1016/0006-2952(87)90440-0

[b45] Daluge S. M. . 1592U89, a novel carbocyclic nucleoside analog with potent, selective anti-human immunodeficiency virus activity. Antimicrobial agents and chemotherapy 41, 1082–1093 (1997). 9145874 10.1128/aac.41.5.1082 PMC163855

[b46] Cox S. W., Aperia K., Albert J. & Wahren B. Comparison of the sensitivities of primary isolates of HIV type 2 and HIV type 1 to antiviral drugs and drug combinations. AIDS research and human retroviruses 10, 1725–1729, doi: 10.1089/aid.1994.10.1725 (1994). 7888232

[b47] Witvrouw M. . Susceptibility of HIV-2, SIV and SHIV to various anti-HIV-1 compounds: implications for treatment and postexposure prophylaxis. Antiviral therapy 9, 57–65 (2004). 15040537

[b48] Pruvost A. . Measurement of intracellular didanosine and tenofovir phosphorylated metabolites and possible interaction of the two drugs in human immunodeficiency virus-infected patients. Antimicrobial agents and chemotherapy 49, 1907–1914, doi: 10.1128/AAC.49.5.1907-1914.2005 (2005). 15855513 PMC1087635

[b49] Ahluwalia G. . Anomalous accumulation and decay of 2′,3′-dideoxyadenosine-5′-triphosphate in human T-cell cultures exposed to the anti-HIV drug 2′,3′-dideoxyinosine. Drug metabolism and disposition: the biological fate of chemicals 21, 369–376 (1993). 8097711

[b50] Parker W. B. . Metabolism of carbovir, a potent inhibitor of human immunodeficiency virus type 1, and its effects on cellular metabolism. Antimicrobial agents and chemotherapy 37, 1004–1009 (1993). 7685993 10.1128/aac.37.5.1004 PMC187879

[b51] Faletto M. B. . Unique intracellular activation of the potent anti-human immunodeficiency virus agent 1592U89. Antimicrobial agents and chemotherapy 41, 1099–1107 (1997). 9145876 10.1128/aac.41.5.1099 PMC163857

[b52] Diamond T. L. . Macrophage tropism of HIV-1 depends on efficient cellular dNTP utilization by reverse transcriptase. The Journal of biological chemistry 279, 51545–51553, doi: 10.1074/jbc.M408573200 (2004). 15452123 PMC1351161

[b53] Kennedy E. M. . Ribonucleoside triphosphates as substrate of human immunodeficiency virus type 1 reverse transcriptase in human macrophages. The Journal of biological chemistry 285, 39380–39391, doi: 10.1074/jbc.M110.178582 (2010). 20924117 PMC2998149

[b54] Stillman B. Deoxynucleoside triphosphate (dNTP) synthesis and destruction regulate the replication of both cell and virus genomes. Proceedings of the National Academy of Sciences of the United States of America 110, 14120–14121, doi: 10.1073/pnas.1312901110 (2013). 23946423 PMC3761580

[b55] Franzolin E. . The deoxynucleotide triphosphohydrolase SAMHD1 is a major regulator of DNA precursor pools in mammalian cells. Proceedings of the National Academy of Sciences of the United States of America 110, 14272–14277, doi: 10.1073/pnas.1312033110 (2013). 23858451 PMC3761606

[b56] Nordlund P. & Reichard P. Ribonucleotide reductases. Annual review of biochemistry 75, 681–706, doi: 10.1146/annurev.biochem.75.103004.142443 (2006). 16756507

[b57] Allouch A. . p21-mediated RNR2 repression restricts HIV-1 replication in macrophages by inhibiting dNTP biosynthesis pathway. Proceedings of the National Academy of Sciences of the United States of America 110, E3997–4006, doi: 10.1073/pnas.1306719110 (2013). 24082141 PMC3801060

[b58] Pauls E. . p21 regulates the HIV-1 restriction factor SAMHD1. Proceedings of the National Academy of Sciences of the United States of America 111, E1322–1324, doi: 10.1073/pnas.1322059111 (2014). 24610778 PMC3986173

[b59] Johnson M. A. & Fridland A. Phosphorylation of 2′,3′-dideoxyinosine by cytosolic 5′-nucleotidase of human lymphoid cells. Molecular pharmacology 36, 291–295 (1989). 2549385

[b60] Beach L. B., Rawson J. M., Kim B., Patterson S. E. & Mansky L. M. Novel inhibitors of human immunodeficiency virus type 2 infectivity. The Journal of general virology 95, 2778–2783, doi: 10.1099/vir.0.069864-0 (2014). 25103850 PMC4233633

[b61] Daly M. B. . Dual anti-HIV mechanism of clofarabine. Retrovirology 13, 20, doi: 10.1186/s12977-016-0254-0 (2016). 27009333 PMC4806454

[b62] De Clercq E. Antiviral agents: characteristic activity spectrum depending on the molecular target with which they interact. Advances in virus research 42, 1–55 (1993). 8430518 10.1016/s0065-3527(08)60082-2

[b63] Donnell D. . Heterosexual HIV-1 transmission after initiation of antiretroviral therapy: a prospective cohort analysis. Lancet 375, 2092–2098, doi: 10.1016/S0140-6736(10)60705-2 (2010). 20537376 PMC2922041

[b64] Lingappa J. R. . Daily acyclovir for HIV-1 disease progression in people dually infected with HIV-1 and herpes simplex virus type 2: a randomised placebo-controlled trial. Lancet 375, 824–833, doi: 10.1016/S0140-6736(09)62038-9 (2010). 20153888 PMC2877592

[b65] Vanpouille C. . Valacyclovir Decreases Plasma HIV-1 RNA in HSV-2 Seronegative Individuals: A Randomized Placebo-Controlled Crossover Trial. Clinical infectious diseases: an official publication of the Infectious Diseases Society of America 60, 1708–1714, doi: 10.1093/cid/civ172 (2015). 25740794 PMC4447783

[b66] Lisco A. . Acyclovir is activated into a HIV-1 reverse transcriptase inhibitor in herpesvirus-infected human tissues. Cell host & microbe 4, 260–270, doi: 10.1016/j.chom.2008.07.008 (2008). 18779052 PMC4210193

[b67] Vanpouille C., Lisco A. & Margolis L. Acyclovir: a new use for an old drug. Curr Opin Infect Dis 22, 583–587, doi: 10.1097/QCO.0b013e32833229b8 (2009). 19726982

[b68] Vanpouille C. . Exploiting the anti-HIV-1 activity of acyclovir: suppression of primary and drug-resistant HIV isolates and potentiation of the activity by ribavirin. Antimicrobial agents and chemotherapy 56, 2604–2611, doi: 10.1128/AAC.05986-11 (2012). 22314523 PMC3346610

[b69] McMahon M. A., Parsons T. L., Shen L., Siliciano J. D. & Siliciano R. F. Consistent inhibition of HIV-1 replication in CD4^+^ T cells by acyclovir without detection of human herpesviruses. Journal of virology 85, 4618–4622, doi: 10.1128/JVI.02423-10 (2011). 21325417 PMC3126274

[b70] Elion G. B. . Selectivity of action of an antiherpetic agent, 9-(2-hydroxyethoxymethyl) guanine. Proceedings of the National Academy of Sciences of the United States of America 74, 5716–5720 (1977). 202961 10.1073/pnas.74.12.5716 PMC431864

[b71] Bonifati S. . SAMHD1 controls cell cycle status, apoptosis and HIV-1 infection in monocytic THP-1 cells. Virology 495, 92–100, doi: 10.1016/j.virol.2016.05.002 (2016). 27183329 PMC4912869

[b72] Tyring S. K., Baker D. & Snowden W. Valacyclovir for herpes simplex virus infection: long-term safety and sustained efficacy after 20 years’ experience with acyclovir. The Journal of infectious diseases 186 Suppl 1, S40–46, doi: 10.1086/342966 (2002). 12353186

[b73] Warnecke S. & Meier C. Synthesis of nucleoside Di- and triphosphates and dinucleoside polyphosphates with cycloSal-nucleotides. The Journal of organic chemistry 74, 3024–3030, doi: 10.1021/jo802348h (2009). 19320463

[b74] Gillis S. & Watson J. Biochemical and biological characterization of lymphocyte regulatory molecules. V. Identification of an interleukin 2-producing human leukemia T cell line. The Journal of experimental medicine 152, 1709–1719 (1980). 6778951 10.1084/jem.152.6.1709 PMC2186024

[b75] Mann D. L. . Origin of the HIV-susceptible human CD4^+^ cell line H9. AIDS research and human retroviruses 5, 253–255, doi: 10.1089/aid.1989.5.253 (1989). 2567177

[b76] Sundstrom C. & Nilsson K. Establishment and characterization of a human histiocytic lymphoma cell line (U-937). International journal of cancer 17, 565–577 (1976). 178611 10.1002/ijc.2910170504

[b77] Tsuchiya S. . Establishment and characterization of a human acute monocytic leukemia cell line (THP-1). International journal of cancer 26, 171–176 (1980). 6970727 10.1002/ijc.2910260208

[b78] Yap M. W., Nisole S., Lynch C. & Stoye J. P. Trim5alpha protein restricts both HIV-1 and murine leukemia virus. Proceedings of the National Academy of Sciences of the United States of America 101, 10786–10791, doi: 10.1073/pnas.0402876101 (2004). 15249690 PMC490012

[b79] Bock M., Bishop K. N., Towers G. & Stoye J. P. Use of a transient assay for studying the genetic determinants of Fv1 restriction. Journal of virology 74, 7422–7430 (2000). 10906195 10.1128/jvi.74.16.7422-7430.2000 PMC112262

[b80] Groom H. C. . Absence of xenotropic murine leukaemia virus-related virus in UK patients with chronic fatigue syndrome. Retrovirology 7, 10, doi: 10.1186/1742-4690-7-10 (2010). 20156349 PMC2839973

[b81] Naldini L. . *In vivo* gene delivery and stable transduction of nondividing cells by a lentiviral vector. Science 272, 263–267 (1996). 8602510 10.1126/science.272.5259.263

[b82] Bainbridge J. W. . *In vivo* gene transfer to the mouse eye using an HIV-based lentiviral vector; efficient long-term transduction of corneal endothelium and retinal pigment epithelium. Gene therapy 8, 1665–1668, doi: 10.1038/sj.gt.3301574 (2001). 11895005

