# Supplementary material for: SAMHD1 enhances nucleoside-analogue efficacy against HIV-1 in myeloid cells
Source: Sci Rep. 2017 Feb 21;7:42824. doi: 10.1038/srep42824 (PMC5318888; doi:10.1038/srep42824)
Supplement: Supplementary Material [file srep42824-s1.pdf]

Supplementary Material

SAMHD1 enhances nucleoside-analogue efficacy against HIV-1 in myeloid cells

Paula Ordonez, Simone Kunzelmann, Harriet C. Groom, Melvyn W. Yap, Simon Weising, Chris. Meier, Kate N. Bishop, Ian A. Taylor and Jonathan P. Stoye

Table S1. Cytotoxicity and anti-HIV-1 activity of nucleoside analogues in T cell lines

Nucleoside analogue	H9			Jurkat		
	CC_{50} (μM)[a] [95% CI][c]	EC_{50} (μM)[b] [95% CI]	SI[d]	CC_{50} (μM) [95% CI]	EC_{50} (μM) [95% CI]	SI
ACV	>200	>200	-	>200	>200	-
GCV	>200	>200	-	>200	>200	-
CFB	0.031 [0.026 - 0.037]	0.094 [0.028 - 0.159]	<1	0.049 [0.025 - 0.073]	0.663 [0.436 - 0.891]	<1
d4T	>200	5.57 [5.33 - 5.82]	>36	>200	6.56 [1.38 - 11.7]	>30
ddI	>200	2.37 [0.803 - 3.94]	>84	>200	1.43 [0.711 - 2.15]	>140
ABC	>200	0.029 [0.022 - 0.036]	>6897	>200	0.026 [0.007 - 0.044]	>7692
NVP	>200	0.158 [0.116 - 0.201]	>1266	>200	0.486 [0.345 - 0.627]	>412
RAL	>200	0.002 [0.002 - 0.003]	>100000	>200	0.004 [0.003 - 0.004]	>50000

[a]CC_{50} is the effective concentration that inhibits cell viability by 50% measured as the percentage of live cells by flow cytometry. Values were calculated by non-linear regression for at least two independent experiments.

[b]EC_{50} is the effective concentration of drug that inhibits infection of cells by 50% measured as percentage of GFP^+ cells. Values were calculated by non-linear regression for at east two independent experiments.

[c] 95% confidence interval. Non-ambiguous values were subject to a replicate test.

[d]SI: Selectivity index, CC_{50}/EC_{50}

Table S2. Cytotoxicity and selectivity index of nucleoside analogues in monocytoid cell lines

Nucleoside analogue	U937				THP-1			
	PMA[-]		PMA[+]		PMA[-]		PMA[+]	
	CC_{50} (μM)[a] [95% CI][b]	SI[c]	CC_{50} (μM) [95% CI]	SI	CC_{50} (μM) [95% CI]	SI	CC_{50} (μM) [95% CI]	SI
ACV	>200	-	>200	>4	>200	-	>200	>304
GCV	>200	-	>200	-	>200	-	>200	>545
CFB	0.090 [0.080 - 0.100]	<1	>1.6	>43	0.059 [0.023 - 0.095]	<1	>1.6	>533
d4T	>200	>31	>200	>28	>200	>32	>200	>51
ddI	>200	>4	>200	>82	>200	>99	>200	>2151
ABC	> 200	>14	>200	>27	>200	>406	>200	>6452
NVP	>200	>396	>200	>1695	>200	>542	>200	>913
RAL	>200	>15385	>200	>3175	>200	>4545	>200	>5405

[a]CC_{50} is the effective concentration of nucleoside analogue that inhibits cell viability by 50% measured as the percentage of live cells by flow cytometry. Values were calculated by non-linear regression from at least two independent experiments.

[b]95% confidence interval. Non-ambiguous values were subject to a replicate test.

[c]SI: Selectivity index, CC_{50}/EC_{50}
